# TXNIP promotes viral replication by disrupting MAVS-mediated antiviral signaling and serves as a therapeutic target for antiviral therapy

**DOI:** 10.1016/j.redox.2025.103756

**Published:** 2025-07-05

**Authors:** Ruilin Chen, Wenyu Wu, Jiajia Han, Qiyun Lei, Jiawan Chen, Dan Guo, Chan Wang, Xiaodong Tang, Min Zou, Shuwen Liu, Xingang Yao

**Affiliations:** a State Key Laboratory of Organ Failure Research, Guangdong Provincial Key Laboratory of New Drug Screening, School of Pharmaceutical Sciences, Southern Medical University, Guangzhou, 510515, PR China; b State Key Laboratory of Ophthalmology, Zhongshan Ophthalmic Center, Sun Yat-sen University, Guangzhou 510060, PR China; c Key Laboratory of Infectious Diseases Research in South China (Southern Medical University), Ministry of Education, Guangzhou, 510515, PR China

**Keywords:** TXNIP, HSV-1, MAVS, Luteolin, innate immune response

## Abstract

Thioredoxin-interacting protein (TXNIP) is a multifunctional regulator involved in oxidative stress, inflammation, and glucose metabolism. In this study, we investigated the role of TXNIP in viral infection and its potential as a therapeutic target. Our findings reveal that HSV-1 infection induces TXNIP upregulation, and elevated TXNIP levels promote viral replication by inhibiting MAVS aggregation. This inhibition disrupts the TBK1/IRF3 signaling pathway, leading to reduced interferon-beta (IFN-β) production and impaired antiviral immunity. Conversely, TXNIP knockout significantly suppresses HSV-1 infection. Furthermore, we utilized Luteolin, a TXNIP degrader previously reported by our group, which restores MAVS aggregation and enhances antiviral responses, demonstrating potent antiviral effects both *in vitro* and *in vivo*. Notably, this mechanism was also validated in dengue virus (DENV2) infection, suggesting a broader role for TXNIP in immune regulation. These findings establish TXNIP as a critical modulator of viral infection and highlight TXNIP degraders as promising candidates for antiviral therapy.

## 1 Introduction

TXNIP (Thioredoxin-interacting protein) is a member of the α-arrestin family, initially identified through its interaction with thioredoxin (TRX) [1]. TXNIP consists of two arrestin domains (N-ARR and C-ARR), two PPxY motifs at the C-terminus, and a leucine (LL) motif involved in endocytosis. Its two highly conserved PPxY motifs, PPCY and PPTY, facilitate protein-protein interactions [2]. TXNIP is a multifunctional protein involved in oxidative stress [3,4], energy metabolism regulation [5,6], tumor suppression [[7], [8], [9]], and the regulation of cellular autophagy [10,11]. Although TXNIP was initially thought to reside exclusively in the cytoplasm, subsequent studies have revealed that under oxidative stress, TXNIP can translocate to various cellular compartments, including mitochondria and the cell surface, potentially contributing to its diverse biological functions [12]. Current studies have demonstrated that TXNIP expression is upregulated under various stimuli [[13], [14], [15]]. Some research suggests that TXNIP upregulation leads to the accumulation of reactive oxygen species (ROS) and the activation of the NLRP3 inflammasome, thereby inducing inflammatory responses [16], which may exert an inhibitory effect on certain viruses. However, studies by Dong Oh Kim et al. indicate that TXNIP directly interacts with TAK1 during bacterial infection and inhibits TAK1 activity by disrupting the formation of the TAK1-Table 1 (TAK1-binding protein 1) complex, consequently suppressing IFN-γ production in NK cells [17]. Additionally, Burton E.M. et al. demonstrated that the interaction between NLRP3 and TXNIP activates the NLRP3 inflammasome, promoting Epstein-Barr virus (EBV) activation, whereas TXNIP knockdown inhibits the release of EBV viral particles [18]. These results imply a potential involvement of TXNIP in virus-induced innate immunity, although direct evidence is currently lacking.

**Table 1:** List of all cloning primers.

Name	Sequence
*Eco*RV-hTXNIP152-F	CCGGATATCATGCCACCCAAGCATTCC
*Xho*I-hTXNIP152-R	CCGCTCGAGAATTCGGGAACATGTATTC
*Eco*RV-hTXNIP300-F	CCGGATATCATGCTGACTCAGAAGTTGTCATC
*Xho*I-hTXNIP300-R	CCGCTCGAGCTGCACATTGTTGTTGAGG
*Kpn*I-MAVS-F	CTTGGTACCATGCCGTTTG
*Xho*I-MAVS180-R	CCGCTCGAGTTAGGACTCCAGGGGGCCAC
*Xho*I-MAVS470-R	CCGCTCGAGTTACACGTGGATCCCAAAGG
*Kpn*I-hMAVS450-F	CGGGGTACCATGGGGCCCTGCCATG
*Kpn*I-hMAVS470-F	CGGGGTACCGTGGCTGAGAACCCCAGC
*Xho*I-hMAVS-R	AGACTCGAGCTAGTGCAGA

The innate immune system recognizes viral components through pattern recognition receptors (PRRs), initiating host antiviral responses. Innate immunity is mediated by cellular proteins induced in response to interferons (IFN-α/β), which plays a central antiviral role in HSV-1 infection [19]. Several signaling pathways, such as cGAS-STING [20] and RIG-I/MAVS [21], have been shown to play pivotal roles in activating type I interferon responses and inducing antiviral defenses. During HSV-1 replication, the cGAS-STING pathway activates effective innate immune responses [22], triggering IRF3 and NF-κB signaling pathways, thereby promoting the secretion of type I interferons and other inflammatory factors [23]. Additionally, the RIG-I/MAVS pathway plays a significant role in the innate immune response to various DNA viruses, including herpesviruses and adenoviruses. Studies have demonstrated that RNA polymerase III (Pol III) can convert poly(dA:dT) into short RNA containing 5′-triphosphate, which is recognized by RIG-I, thereby generating RIG-I ligands from dsDNA and acting upstream of RIG-I [24,25]. Double-stranded RNA (dsRNA) produced during HSV-1 replication can be recognized by intracellular TLR3 or RLRs, activating IRF3 signaling and promoting the secretion of IFN-I (IFN-α/β) and other inflammatory factors [26]. Targeting these pathways not only holds promise for improving the efficacy of existing drugs but also provides a direction for identifying new antiviral targets.

Beyond DNA virus infections, innate immune responses also play a critical role in RNA virus infections [27], including those caused by SARS-CoV-2 and dengue virus (DENV). Also, RNA viruses are recognized by PRRs, which activate the RIG-I/MAVS signaling pathway, leading to phosphorylation of IRF3 and subsequent secretion of type I interferons [27]. This signaling cascade promotes an antiviral state in infected cells. DENV, a single-stranded positive-sense RNA virus transmitted by mosquitoes in tropical and subtropical regions, can cause severe, life-threatening conditions such as dengue hemorrhagic fever (DHF) and dengue shock syndrome (DSS), with high mortality rates. Currently, there are no specific antiviral drugs available for dengue [28]. Therefore, investigating the relationship between TXNIP and the innate immune response to viruses could provide novel therapeutic strategies for combating both DNA and RNA viral infections.

Currently, the regulatory role of TXNIP in viral infections, particularly in HSV-1 or DENV infection, remains poorly understood, making it essential to deeply explore its specific regulatory mechanisms. Based on the aforementioned research background and current advancements, this study mainly uses HSV-1 infection as a model system to investigate whether TXNIP influences HSV-1 infection and to clarify its underlying mechanisms, while also aiming to systematically evaluate the relationship between TXNIP and the body's innate immunity in DNA and RNA virus infections by using the common DNA virus HSV-1 and the RNA virus DENV, a representative commonly used in our laboratory.

## 2 Materials and methods

### 2.1 Materials

DMEM medium were purchased from Thermo Fisher (USA). FBS were purchased from Excell (FSD500, China). Primers were synthesized by Tsingke Biotechnology (China). Restriction endonucleases, T4 Polynucleotide Kinase, and T4 DNA Ligase were purchased from NEB (USA). The human alpha herpesvirus 1 strain F (HSV-1) was a gift from Guangzhou Institutes of Biomedicine and Health. HSV-GFP strain was presented by Professor Zhou Pingzheng from School of Pharmacy, Southern Medical University. The New Guinea strain of Dengue Virus 2 (DENV2) was provided by Professor Chen Xiaoguang of Southern Medical University. Luteolin was purchased from TargetMol (T1027, China) with a purity of above 98 %.

### 2.2 Cell culture

293T cells were purchased from the National Collection of Authenticated Cell Cultures (China). 293T cells and HeLa cells were cultured in DMEM supplemented with 10 % FBS and 100 U/mL penicillin and streptomycin.

### 2.3 Plasmid construction

Plasmids expressing human TXNIP-Flag were purchased from Miaoling Bio (P37969, China). MAVS truncations were all amplified by PCR and connected to the vector of pcDNA3.1-HA, and the truncated plasmids of TXNIP were all amplified by PCR and connected to the vector of pcDNA3.1-Flag. These shRNA plasmids used for gene knockdown in this study were synthesized by Guangzhou Jidan Bio, utilizing the pLKO.1 vector backbone template. The PCR primers used are shown in the following table. All plasmid constructs were confirmed by DNA sequencing. For transient transfection of plasmids into 293T and HeLa cells, Lipofectamine 3000 (Invitrogen, L3000015, USA) reagent was used.

### 2.4 Plasmid amplification

DNA was transformed and amplified by heat shock method. Plasmid DNA or recombinant DNA adheres to the surface of bacterial cells, and after a short period of heat shock treatment at 42 °C, it promotes the absorption of DNA. And then competent cells grow in a medium containing antibiotic. After 12–16 h, a single colony was picked out and cultured from 2 mL to 400 mL in turn. According to the manufacturer's instructions, the plasmid extraction was completed by using the MN NucleoBond Xtra Midi transfection plasmid extraction kit (740410.50, MN, Germany). Escherichia coli DH5α competent cells were purchased from TAKARA (9057, Japan), and Stbl3 competent cells were made in the laboratory.

### 2.5 Plasmid transfection

293T cells or HeLa cells were seeded in 24-well plates. Dilute 300 ng plasmid with 50 μL culture medium and 1 μL Lipo 3000 reagent with 50 μL culture medium in each well, incubate them for 5 min, then mix them evenly, incubate them at room temperature for 15 min, then slowly drop them into cells and shake them evenly. Follow-up treatment with or without virus infection was carried out after 24 h.

### 2.6 Virus infection *in vitro*

HSV-1 and DENV2 were used to infect cells in the experiment. After being infected with virus in serum-free medium for 1 h, the culture supernatant was collected, and the cells were continuously cultured in the medium containing 2 % fetal bovine serum (FBS). Total cells were collected at specified time points for RT-qPCR and Western blotting.

### 2.7 Antiviral assay *in vivo*

All experimental procedures were executed according to the protocols approved by the Institutional Animal Care and Use Committee of the Southern Medical University. All mice were obtained from the Experimental Animal Center of Southern Medical University (Guangzhou, Guangdong), and strictly maintained a 24-h reverse light/dark cycle, providing food and water at will. After adaptation, 6-week-old male BALB/c mice were randomly assigned into several groups, including control (Control, n = 5), HSV-1 infected (n = 5), HSV-1 + Luteolin (50 mg/kg) (n = 5), and Luteolin (50 mg/kg) (n = 5). The virus was diluted to 1 × 10^7^ PFU/mL with serum-free DMEM. Mice (n = 5) received 20 μL of nasal treatment while anesthetized by isoflurane inhalation (2.5 %–5.0 %). After infection, mice were given the continuous gavage administration of Luteolin for 7 days. Weight loss was monitored daily, and mice were sacrificed when weight loss reached 20 %, and the tissues were isolated for further analysis. The disease severity was determined with the following scoring analyse [29,30], which distinguished in (I) body weight loss, (II) fur condition, (III) ocular symptoms and (IV) behavior and other disease symptoms. (I) 0 (<5 % weight loss), 1 (5–10 %), 2 (10–20 %), or 3 (>20 %) points for weight changes; (II) 0 (smooth), 1 (Dull-haired), 2 (sparse-haired), or 3 (severe alopecia) points for fur condition; (III) 0 (normal), 1 (unilateral mild swelling), 2 (bilateral swelling or unilateral severe inflammation), or 3 (bilateral ulceration or eye rupture) points for ocular symptoms; (IV) 0 (normal), 1 (sluggish), 2 (unsteady gait), or 3 (paralysis) points for behavior and other disease symptoms.

### 2.8 Real-time fluorescence quantitative PCR (RT-qPCR)

Total RNA of cells was extracted with RNA extraction kit (Vazyme, cell/tissue total RNA extraction kit RC101, China), and RNA was reverse transcribed into cDNA with reverse transcription premix (Vazyme, R323-01, China). According to the manufacturer's instructions, the reverse transcription products of different samples were amplified by LightCycler 480 software (Roche) using SYBR Green PCR Master Mix (Vazyme, Q711, China), and the data were standardized into the expression of gene glyceraldehyde-3-phosphate dehydrogenase (GAPDH) in each individual sample. 2^−△△Ct^ method was used to calculate relative expression changes.

### 2.9 Western blotting

To collect total cellular protein, the treated cell samples were washed with cold PBS. Subsequently, 50 μL of RIPA lysis buffer (KeyGEN BioTECH, KGP703, China) supplemented with 100-times phosphatase inhibitor (Jiangsu KeyGEN BioTECH, KGP602, China) and 1000-times protease inhibitor (Jiangsu KeyGEN BioTECH, KGP603, China) was added to each well of a 24-well plate. The cells were then scraped and collected into a 1.5 mL Eppendorf tube. An equal volume of 2 × loading buffer was added, mixed thoroughly, and the samples were denatured at 105 °C for 10 min. A 10 % SDS-polyacrylamide gel was prepared, and an equal amount of protein was loaded into each well for electrophoresis. Electrophoresis was initially performed at a constant voltage of 80V for 25 min, followed by an increase to 120V. The duration of electrophoresis was determined based on the separation of the pre-stained protein marker and the migration of bromophenol blue. After electrophoresis, proteins were transferred to a membrane at 100V for 90 min. The membrane was blocked with 5 % (w/v) skim milk at room temperature for 1 h, followed by incubation with primary and secondary antibodies. Finally, the membrane was treated with ECL developer A/B solution (NCM Biotech, P10060, China) in a 1:1 ratio, and the signal was detected by exposure and development.

### 2.10 Luciferase reporter assay

293T cells were seeded in a 24-well plate (1.5 × 10^5^/mL), transfected with IFN-Beta-Luc or ISRE-Luc plasmid 50 ng after 24 h, changed the culture medium after 8 h, followed up with infection treatment after 24 h, discarded the supernatant after 48 h, and washed with PBS. 200 μL of Luciferase cell culture Lysis (Promega, E1531, USA) was added to each well for lysis, and the cell lysate supernatant was shaken at room temperature for 20 min on a shaker, and 40 μL of cell lysate supernatant was absorbed into a 96-well whiteboard, with 3 wells for each sample. 40 μL of luciferase substrate (Promega, E1501, USA) diluted with ddH_2_O1: 1(v/v) was added to each well, and the Luciferase level was detected in a multifunctional microplate detection system (M1000PRO, Switzerland).

### 2.11 Co-immunoprecipitation assay (Co-IP)

293T cells were seeded in a 6-well plate (3 × 10^5^/mL). After 24 h, TXNIP-Flag and MAVS-HA plasmids were transfected with 1.5 μg each. After 8 h, the medium in the cells was removed. After 48 h, the cells were washed once with PBS, and the precooled IP lysate was added for ice lysis for 10 min. Cell lysate was collected at 13000×*g* and centrifuged for 10 min to precipitate cell fragments. Incubate IP beads with Flag antibody (Sigma, F1804-50UG, USA) or HA antibody (CST, 3724S, USA) in advance at 4 °C overnight, and then clean the beads. Add the supernatant of the collected cell lysate and incubate overnight at 4 °C. After taking out the mixture of antibody, cell lysate and beads, IP buffer was washed for three times, and 50 μL of 2 × loading buffer (containing 100 mM Tris-HCl pH 6.8, 4 % (w/v) SDS, 20 % (v/v) glycerol, 0.2 % (w/v) bromophenol blue and 1 % (v/v) β-mercaptoethanol was added. The immune precipitate was eluted by incubation at 105 °C for 10 min, followed by storage at −20 °C.

### 2.12 Immunofluorescence assay

293T cells were seeded in a confocal dish (5 × 10^4^/mL). After 24 h of culture, 1 μg of TXNIP-Flag and MAVS-HA plasmids were transfected. After 8 h, the medium in the cells was removed after 48 h. After PBS washing, 1 mL of 4 % paraformaldehyde was added and fixed at room temperature for 30 min, and 0.1 %Triton was used for 20 min, and 3 %BSA was used for 1 h. Incubate with an anti-Flag antibody or HA antibody at 4 °C overnight. PBS was washed and combined with fluorescent secondary antibody at room temperature for 1 h 488 donkey anti-rabbit IgG(H + L) high cross-adsorption secondary antibody and 568 goat anti-mouse IgG(+L) cross-adsorption secondary antibody were purchased from Invitrogen (A21206, A11004, USA). The nucleus was stained with Hoechst (Jiangsu KeyGEN BioTECH, KGR0001-1, China). Images were taken using an inverted laser confocal microscope (LSM 880 with Airyscan, ZEISS, Germany).

### 2.13 Bimolecular fluorescent complimentary (BiFC)

293T cells were seeded in a confocal dish (5 × 10^4^/mL), cultured for 24 h, then transfected with pBiFC-humanTXNIP-VN173 and pBiFC-hMAVS-VC155 1000 ng as previous report [31], changed the solution after 8 h, removed the medium in the cells after 48 h, and washed with PBS. Images were taken using an inverted laser confocal microscope (LSM 880 with Airyscan).

### 2.14 Generation of knockout cell lines by CRISPR/Cas9

The gene editing with CRISPR/Cas9 system was used to produce TXNIP-KO cells. The sequence of TXNIP-sgRNA was as follows: F: CACCGCCATCTCATTCCACCTGAA, R: AAACTTCAGTGAGAGATGGC. 293T cells were seeded in a 24-well plate (1.5 × 10^5^/mL), transfected with TXNIP-sgRNA plasmid 250 ng, changed the culture medium after 8 h. Puromycin (Beyotime, ST551, China) was added for screening after 48h. Monoclonal cells were selected from the surviving cells screened by puromycin, and the used TXNIP-KO cells were verified by DNA sequencing, Western blotting and RT-qPCR.

### 2.15 Semi-denaturing detergent agarose gel electrophoresis

293T cells were seeded in a 24-well plate (1.5 × 10^5^/mL), transfected with pcDNA3.1a-vector or TXNIP-Flag after 24 h, changed the solution after 8 h, removed the culture medium in the cells after 48 h, and cultured in 1 × buffer (0.5 × TBE, 10 % glycerol, 2 % SDS and 0.0020 bromophenol blue), and loaded onto a vertical 1.5 % agarose gel (Bio-Rad, USA). Electrophoresis at 90V in electrophoresis buffer (0.5 × TBE and 0.1 % SDS) at 4 °C for 35 min, 100V for 90 min and then transferred to PVDF membrane for further Western blotting.

### 2.16 Primary mouse blood monocytes isolation

Blood monocytes were isolated from mice via retro-orbital bleeding using mouse lymphocyte separation solution (Dakewe Biotech, 7211011, China) through density gradient centrifugation (800×*g*, 25 min, room temperature) [32]. After two washes with PBS, the cells were resuspended in complete RPMI-1640 medium (supplemented with 10 % FBS and 1 % penicillin/streptomycin), counted with a hemocytometer, and seeded at a density of 2 × 10^6^ cells/mL. The monocytes were then infected with HSV-1 or DENV2 infection. Viral replication and TXNIP protein expression were analyzed at indicated time points post-infection.

### 2.17 Statistical analysis

Statistical analysis was performed using GraphPad Prism software (version 9, GraphPad). Statistical significance was determined via two-way analysis of variance (ANOVA), and one-way ANOVA was performed to compare multiple samples. Statistical significance between groups was determined by two-tailed Student's *t*-test. All the data are expressed as the Mean ± standard deviation (SD). P value < 0.05 was regarded as statistically significant.

## 3 Results

### 3.1 TXNIP is upregulated by HSV-1 infection and promotes viral replication

**Fig. 1:**
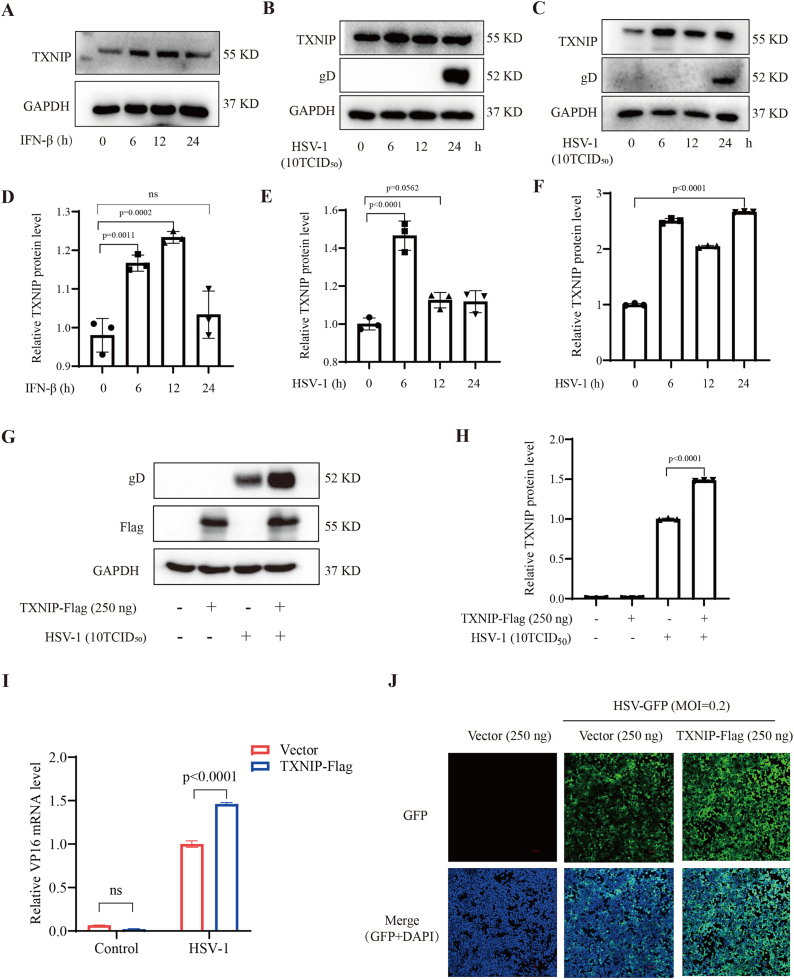
TXNIP is upregulated by HSV-1 infection and promotes viral re**plication.** (A) Western blotting was performed to assess TXNIP protein expression levels in 293T cells after stimulation with IFN-β at 0, 6, 12, and 24 h. (B) TXNIP protein expression levels in 293T cells were detected by Western blotting after infection with HSV-1 at 0, 6, 12, and 24 h. (C) TXNIP protein expression levels in blood monocytes cell were detected by Western blotting after infection with HSV-1 at 0, 6, 12, and 24 h. (D) Quantitative TXNIP protein levels analysis of (A). (E) Quantitative TXNIP protein levels analysis of (B). (F) Quantitative TXNIP protein levels analysis of (C). (G) 293T cells were transfected with either an empty vector (pcDNA3.1a) or a TXNIP-Flag plasmid for 24 h, followed by infection with HSV-1 for an additional 24 h. The expression level of the viral protein gD was subsequently assessed by Western blotting. (H) Quantitative analysis of gD protein expression levels. (I) The expression level of VP16 mRNA in both groups was measured by RT-qPCR. (J) Fluorescence microscopy images of 293T cells transfected with either the empty vector (pcDNA3.1a) or the TXNIP overexpression plasmid, followed by infection with HSV-GFP for 24 h. Data are represented as Mean ± SD (n = 3,p < 0.05 was considered as a statistical difference). Statistical significance was determined by one-way ANOVA or two-sided Student's t-test as appropriate.

The expression levels of TXNIP were first assessed after stimulation with IFN-β cytokines and HSV-1 infection. Subsequently, the impact of TXNIP on viral replication was examined through overexpression and knockdown experiments. 293T cells were treated with IFN-β cytokines at different time points (0, 6, 12, and 24 h), and cellular protein and RNA were harvested after 24 h. Results from Western blotting and RT-qPCR analyses showed that TXNIP expression was significantly enhanced following IFN-β stimulation (Fig. 1A and D), with corresponding increases in mRNA levels (Fig. S1A). Similar observations were made in 293T cells infected with HSV-1 at various time points through Western blotting (Fig. 1B and E) or RT-qPCR (Fig. S1B). We also validated this in primary mouse blood monocytes and found that HSV-1 infection upregulated TXNIP, which is consistent with the results at the cell line level (Fig. 1C and F). These results indicate that TXNIP expression is upregulated in response to HSV-1 infection.

To investigate the role of TXNIP in HSV-1 infection, a TXNIP overexpression plasmid was transfected into 293T cells, followed by HSV-1 infection. Western blotting and RT-qPCR analyses revealed that TXNIP overexpression significantly increased the expression of the viral glycoprotein gD (Fig. 1G-H), along with a marked upregulation of VP16 mRNA levels (Fig. 1I). Additionally, HSV-GFP infection assays confirmed that TXNIP overexpression enhances HSV-1 infectivity (Fig. 1J). These results collectively highlight the promotive effect of TXNIP on HSV-1 infection. Consistent findings were also observed in DENV2-infected cells, where dengue virus infection promoted TXNIP protein expression as early as 6 h post-infection in primary mouse blood monocytes cells (Fig. S2A–S2B). Meanwhile, TXNIP overexpression similarly enhanced viral protein expression (Fig. S2C–S2D) and gene transcription (Fig. S2E), further supporting the conserved role of TXNIP in facilitating viral pathogenesis across different virus families. Together, these data suggest that TXNIP may serve as a potential therapeutic target for antiviral intervention.

### 3.2 Knockout of the *TXNIP* gene inhibits HSV-1 infection

**Fig. 2:**
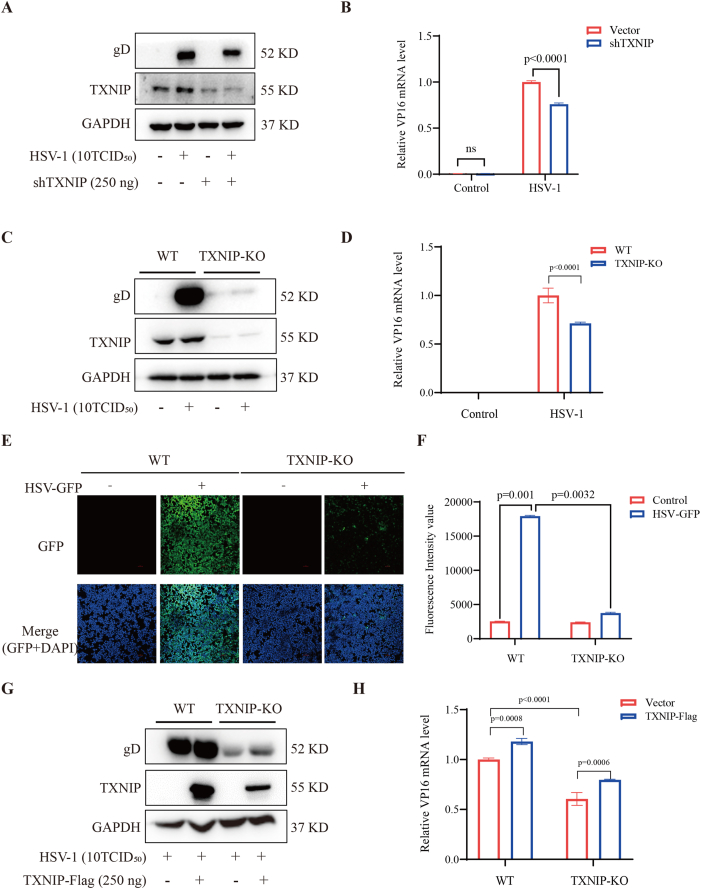
Knockout of the TXNIP gene inhibits HSV-1 **infection.** (A) 293T cells were transfected with either an empty vector (pLKO.1 vector) or TXNIP-shRNA. After 24 h, the cells were infected with HSV-1 for an additional 24 h, and the expression levels of gD and TXNIP proteins were assessed by Western blotting. (B) The transcription level of VP16 mRNA under the same conditions as in (A) was measured by RT-qPCR. (C) 293T-WT and TXNIP-KO cells were infected with HSV-1, and after 24 h, RNA and proteins were extracted. The expression of the viral protein gD was detected by Western blotting. (D) The mRNA expression of the viral gene VP16 was quantified by RT-qPCR. (E) 293T-WT and TXNIP-KO cells were infected with HSV-GFP, and fluorescence images were captured using a fluorescence microscope after 24 h. (F) The right figure is the fluorescence intensity value. (G) Following TXNIP or vector control (pcDNA3.1a) overexpression in both 293T-WT and TXNIP-KO cells, the cells were infected with HSV-1, and the expression level of the viral protein gD was assessed by Western blotting. (H) Under the same conditions as in (G), the mRNA level of the viral gene VP16 was quantified using RT-qPCR. Data are represented as Mean ± SD (n = 3,p < 0.05 was considered as a statistical difference). Statistical significance was determined by two-sided Student's t-test.

Given that TXNIP overexpression promotes HSV-1 infection, we further investigated the functional role of TXNIP by silencing its expression. A TXNIP-specific shRNA plasmid (pLKO.1-TXNIP-shRNA) was synthesized and transfected into 293T cells. Knockdown of TXNIP significantly inhibited HSV-1 infection, as evidenced by a notable reduction in the expression of the viral glycoprotein gD (Fig. 2A) and a marked decrease in VP16 mRNA levels compared to the control group transfected with an empty vector (Fig. 2B). To further elucidate the relationship between TXNIP and HSV-1 infection, we designed three sgRNAs targeting TXNIP and identified TXNIP-sgRNA32 as the most efficient based on T7 endonuclease I (T7EI) assay results (Fig. S1C). Using CRISPR-Cas9-mediated gene editing, we successfully generated a TXNIP knockout (TXNIP-KO) 293T cell line. The knockout was confirmed by significantly reduced TXNIP mRNA (Fig. S1D) and protein levels (Fig. S1E–S1F) compared to wild-type (WT) cells. Following HSV-1 infection, TXNIP-KO cells exhibited substantially lower expression of the viral protein gD and decreased VP16 mRNA levels compared to WT cells (Fig. 2C-D). Furthermore, HSV-GFP infection assays showed a marked reduction in fluorescence intensity in TXNIP-KO cells, indicating impaired viral replication (Fig. 2E-F). These results demonstrate that TXNIP deletion effectively suppresses HSV-1 infection.

To validate this finding, we reintroduced TXNIP into both WT and TXNIP-KO cells and subsequently infected the cells with HSV-1. In TXNIP-KO cells, TXNIP overexpression restored gD protein expression (Fig. 2G) and significantly increased VP16 mRNA levels (Fig. 2H), reversing the inhibitory effects of TXNIP deletion. These data further confirm the critical role of TXNIP in facilitating HSV-1 infection. Consistent results were observed in DENV2-infected cells, where TXNIP deletion similarly suppressed viral replication, indicated by viral protein level (Fig. S2F–S2G) and mRNA level (Fig. S2H). Knockdown of TXNIP using shRNA also reduced the viral NS3 protein level, which was consistent with the results observed in TXNIP-KO cells (Fig. S2I). Furthermore, overexpression of TXNIP in TXNIP-KO cells reversed the antiviral effect induced by TXNIP knockout (Fig. S2J). Collectively, these findings suggest that TXNIP is a positive regulator of both HSV-1 and DENV2 replication and may serve as a potential antiviral target.

### 3.3 TXNIP overexpression inhibited the TBK1/IRF3 pathway activation

**Fig. 3:**
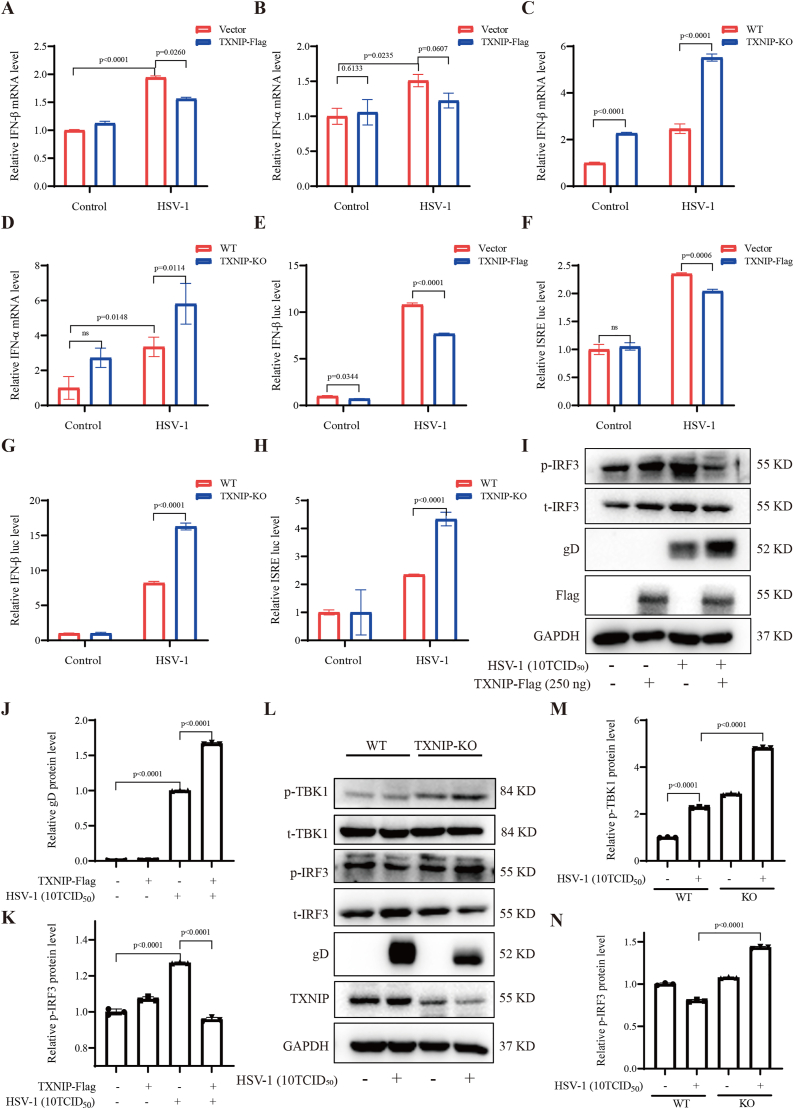
TXNIP overexpression inhibited the TBK1/IRF3 pathway a**ctivation.** (A–B) 293T cells were transfected with either an empty vector (Vector) or TXNIP-Flag for 24 h, followed by HSV-1 infection for an additional 24 h. The mRNA levels of IFN-β and IFN-α were measured by RT-qPCR. (C–D) 293T-WT and TXNIP-KO cells were infected with HSV-1, and after 24 h, the mRNA levels of IFN-β and IFN-α were detected by RT-qPCR. (E) 293T cells were co-transfected with TXNIP-Flag and IFN-β-luc plasmids, followed by HSV-1 infection 24 h later. Luciferase intensity was measured 24 h post-infection. (F) 293T cells were co-transfected with TXNIP-Flag and ISRE-luc plasmids, followed by HSV-1 infection 24 h later. Luciferase intensity was measured 24 h post-infection. (G–H) 293T-WT and TXNIP-KO cells were transfected with IFN-β-luc and ISRE-luc plasmids, respectively, infected with HSV-1, and luciferase intensity was measured 24 h post-infection. (I) Under the same conditions as in (A), cell lysates were collected, and the levels of *p*-IRF3/t-IRF3 were analyzed by Western blotting. (J) Quantitative results of gD protein of (I). (K) Quantitative results of *p*-IRF3/t-IRF3 levels of (I). (L)Under the same conditions as in (C), cell lysates were collected, and the levels of *p*-IRF3/t-IRF3 and *p*-TBK1/t-TBK1 were analyzed by Western blotting. (M) Quantitative *p*-TBK1/t-TBK1 protein levels analysis of (L). (N) Quantitative results of *p*-IRF3/t-IRF3 levels of (L). Data are represented as Mean ± SD (n = 3,p < 0.05 was considered as a statistical difference). Statistical significance was determined by two-sided Student's t-test.

IFN-β plays a central role in the host antiviral immune response, and its expression level serves as an indicator of the antiviral state of host cells. To investigate the effect of TXNIP on type I interferon responses during viral infection, we examined the expression levels of IFN-β and IFN-α following HSV-1 infection. 293T cells were transfected with either an empty vector (Vector) or a TXNIP-Flag plasmid, followed by HSV-1 infection 24 h post-transfection. Total RNA was extracted 24 h after infection, and mRNA levels of IFN-β and IFN-α were quantified by RT-qPCR. Compared to the control group, TXNIP overexpression led to a modest reduction in the mRNA expression of both IFN-β and IFN-α (Fig. 3A–B). Conversely, when comparing wild-type (293T-WT) and TXNIP knockout (TXNIP-KO) cells infected with HSV-1, TXNIP-KO cells displayed a slight increase in IFN-β and IFN-α mRNA levels relative to WT cells (Fig. 3C-D).

To further assess the functional impact of TXNIP on type I interferon signaling, a luciferase reporter assay was employed to evaluate IFN-β promoter activity. The results demonstrated that TXNIP overexpression slightly suppressed IFN-β promoter-driven luciferase activity following HSV-1 infection (Fig. 3E-F), while TXNIP-KO cells exhibited elevated luciferase activity in response to HSV-1 infection (Fig. 3G-H). Subsequent analysis of key signaling molecules in the IFN pathway revealed that TXNIP overexpression reduced the ratio of phosphorylated IRF3 (*p*-IRF3) to total IRF3 (t-IRF3) following HSV-1 infection (Fig. 3I and K). In contrast, TXNIP-KO cells showed a moderate increase in both the *p*-IRF3/t-IRF3 and *p*-TBK1/t-TBK1 ratios (Fig. 3L-N), suggesting enhanced activation of the IFN signaling cascade. These findings diverge from the classical role of TXNIP in promoting antiviral responses via the NLRP3 inflammasome [33] and instead suggest that, under HSV-1 infection conditions, TXNIP functions as a negative regulator of type I interferon responses. Collectively, these data indicate that TXNIP suppresses the host antiviral immune response during HSV-1 infection.

### 3.4 TXNIP directly interact with MAVS

The above results indicate that TXNIP reduces IFN-β production during HSV-1 infection, which suggests that TXNIP may play a regulatory role in innate immune responses, particularly in the IFN-β signaling pathway, under HSV-1 infection. Given the critical role of IFN-β in antiviral immunity, we further explored the molecular mechanisms by which TXNIP regulates IFN-β production. We hypothesized that TXNIP might modulate downstream immune responses by interacting with key components of the IFN-β signaling pathway, such as MAVS, TBK1, IRF3, cGAS or STING. To test this hypothesis, we investigated the potential interactions between TXNIP and several key molecules in the IFN-β signaling pathway, including MAVS, TBK1, IRF3, cGAS and STING. These molecules are essential for IFN-β production triggered by viral infection or other immune stimuli. MAVS, and STING, an adaptor protein downstream of RLRs, converge at TBK1, which phosphorylates IRF3 to promote its dimerization, nuclear translocation, and subsequent induction of IFN-β gene expression. In this study, we aimed to determine whether TXNIP physically interacts with MAVS, TBK1, IRF3, cGAS or STING and whether these interactions are involved in regulating IFN-β production.

**Fig. 4:**
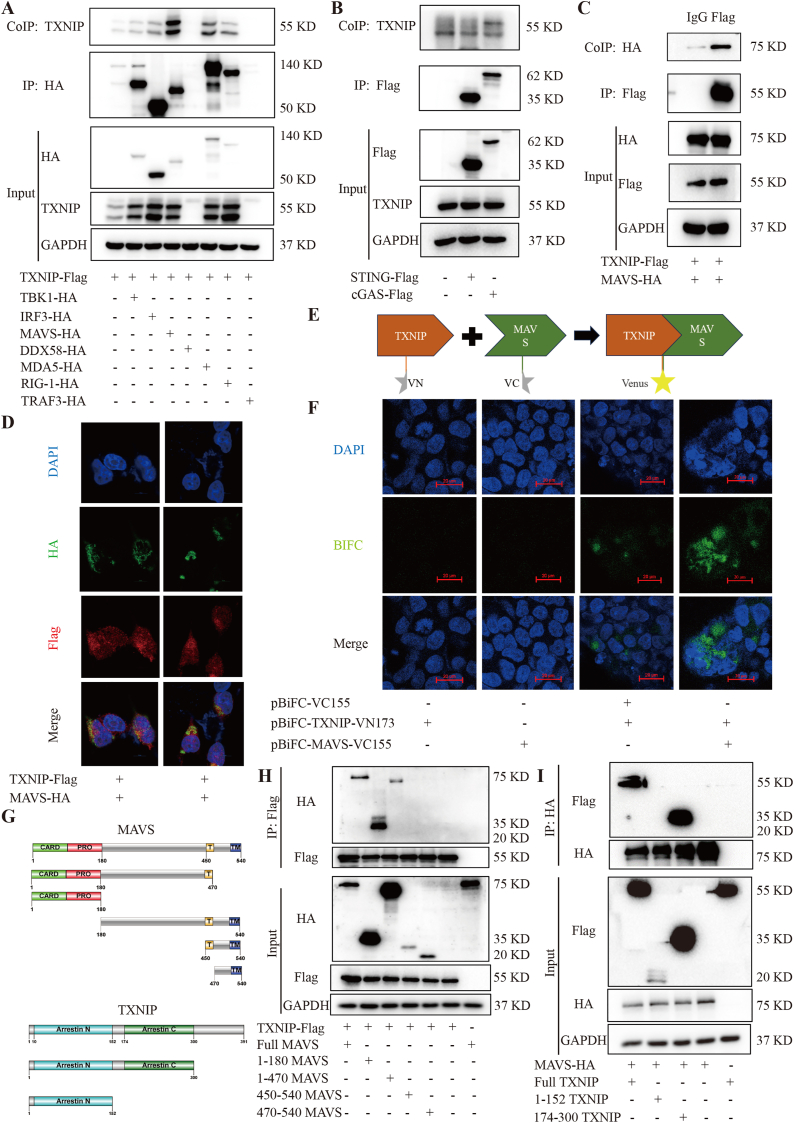
TXNIP directly interact with MAVS. (A–B) Co-immunoprecipitation (Co-IP) analysis of TXNIP with TBK1, IRF3, MAVS, MDA5, DDX58, RIG-I, STING, and cGAS in 293T cells. The lower band represents the endogenous TXNIP in cells, while the upper band is the overexpressed TXNIP-flag band. (C) Immunoprecipitation of HA-tagged MAVS using an anti-Flag (TXNIP) antibody in 293T cells. (D) Representative confocal microscopy images showing co-localization of TXNIP-Flag and MAVS-HA in 293T cells. (E–F) Bimolecular fluorescence complementation (BiFC) assay demonstrating the interaction between TXNIP-Flag and MAVS-HA in 293T cells. (G) The schematic diagram of the domains of TXNIP and MAVS. (H–I) Co-IP analysis of the interaction between TXNIP-Flag truncation and MAVS-HA truncation in 293T cells. All the experiments were independently repeated three times.

First, we obtained expression plasmids for TBK1, IRF3, MAVS, MDA5, DDX58, RIG-I, STING and cGAS and transfected them into 293T cells. Immunoprecipitation using anti-Flag or anti-HA antibodies revealed that TXNIP interacts with MAVS (Fig. 4A-B). To confirm this interaction, we used an anti-Flag (TXNIP) antibody to immunoprecipitate HA-tagged MAVS (Fig. 4C), demonstrating a direct interaction between TXNIP and MAVS. Furthermore, immunofluorescence assay showed clear co-localization of TXNIP (Flag) and MAVS (HA) (Fig. 4D). The interaction between TXNIP and MAVS was further validated using a bimolecular fluorescence complementation (BiFC) assay (Fig. 4E-F). In this assay, the fluorescent protein Venus was split into two non-fluorescent fragments, VN173 and VC155, which were fused to TXNIP and MAVS, respectively. Upon interaction, the fragments reconstituted an active Venus fluorescent protein, producing fluorescence. Additionally, by constructing truncated variants of TXNIP and MAVS (Fig. 4G), we identified that the 174–300 fragment of TXNIP interacts with the 1–180 fragment of MAVS (Fig. 4H-I). These findings provide strong evidence for a direct interaction between TXNIP and MAVS, suggesting that TXNIP may influence HSV-1 infection by modulating MAVS function.

### 3.5 TXNIP inhibits MAVS aggregation

Given the confirmed direct physical interaction between TXNIP and MAVS-validated through Co-IP and BiFC assays, we next investigated the functional implications of this interaction. As MAVS is a central adaptor in the RLR signaling pathway, we hypothesized that TXNIP may regulate downstream antiviral responses by modulating MAVS aggregation or activation. MAVS aggregation is a critical step in its activation and downstream signal transduction. Upon viral detection, MAVS oligomerizes to form high-molecular-weight aggregates on the mitochondrial membrane, which serve as platforms for the recruitment and activation of downstream effectors such as TBK1 and IRF3, ultimately leading to the induction of IFN-β expression [34]. Thus, the aggregation state of MAVS directly influences the strength and efficiency of the innate immune response.

**Fig. 5:**
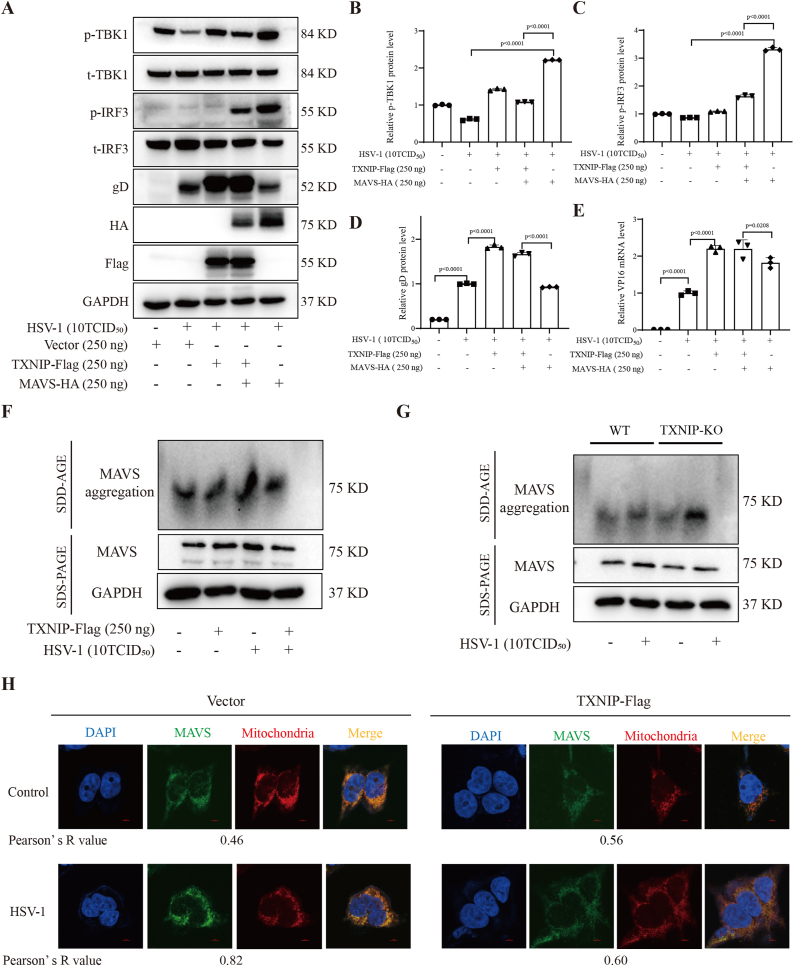
TXNIP inhibits MAVS aggregation. (A) 293T cells were transfected or co-transfected with TXNIP and MAVS, followed by HSV-1 infection. The levels of gD protein, *p*-TBK1, and *p*-IRF3 were detected by Western blotting. (B–D) Quantitative *p*-TBK1/t-TBK1, *p*-IRF3/t-IRF3, and gD protein levels analysis of (A). (E) Under the same conditions as in (A), the mRNA level of VP16 was measured by RT-qPCR. (F) Western blotting analysis of MAVS aggregation in TXNIP-overexpressing 293T cells infected with HSV-1, using semi-denaturing detergent agarose gel electrophoresis (SDD-AGE, top) and SDS-PAGE (bottom), at 24 h. (G) Western blotting analysis of MAVS aggregation in 293T-WT and TXNIP-KO cells infected with HSV-1, using SDD-AGE (upper panel) and SDS-PAGE (lower panel), at 24 h. (H) Immunofluorescence staining was performed on HSV-1-stimulated 293T cells with TXNIP overexpression, and mitochondrial MAVS aggregation was analyzed by confocal microscopy 24 h post-HSV-1 infection. Data are represented as Mean ± SD (n = 3,p < 0.05 was considered as a statistical difference). Statistical significance was determined by one-way ANOVA.

To determine whether TXNIP regulates MAVS aggregation, we first co-expressed TXNIP and MAVS in 293T cells, followed by HSV-1 infection. After 24 h, cell lysates were analyzed for gD protein levels and phosphorylation of TBK1 and IRF3 (Fig. 5A). Overexpression of MAVS alone resulted in reduced gD protein levels, indicating enhanced antiviral activity. However, co-expression of TXNIP reversed this effect, significantly increasing gD levels, suggesting that TXNIP suppresses MAVS-mediated antiviral responses. Consistently, MAVS overexpression increased the *p*-TBK1/t-TBK1 and *p*-IRF3/t-IRF3 ratio, while co-expression with TXNIP reduced TBK1 and IRF3 activation (Fig. 5B-D).

In parallel, RT-qPCR analysis of VP16 mRNA levels revealed that TXNIP co-expression significantly elevated viral gene expression compared to MAVS overexpression alone (Fig. 5E). To directly assess MAVS aggregation, we performed semi-denaturing detergent agarose gel electrophoresis (SDD-AGE). The results showed that TXNIP overexpression markedly reduced MAVS aggregate formation (Fig. 5F). Conversely, TXNIP knockout enhanced MAVS aggregation in response to viral infection (Fig. 5G), further supporting its inhibitory role. Additionally, immunofluorescence assays demonstrated that TXNIP overexpression diminished MAVS aggregation and its mitochondrial co-localization following HSV-1 infection (Fig. 5H). The inhibitory effects of TXNIP on MAVS aggregation were confirmed in the context poly(I:C) stimulation (Fig. S3A-S3D), a well known MAVS activator. Taken together, these results indicate that TXNIP negatively regulates MAVS aggregation during viral infection, thereby attenuating activation of downstream signaling pathways such as TBK1 and IRF3. This modulation likely contributes to the suppression of IFN-β production and a weakened innate immune response, highlighting TXNIP as a potential regulatory node in host-virus interactions.

### 3.6 TXNIP degraders significantly inhibit viral replication *in vitro*

**Fig. 6:**
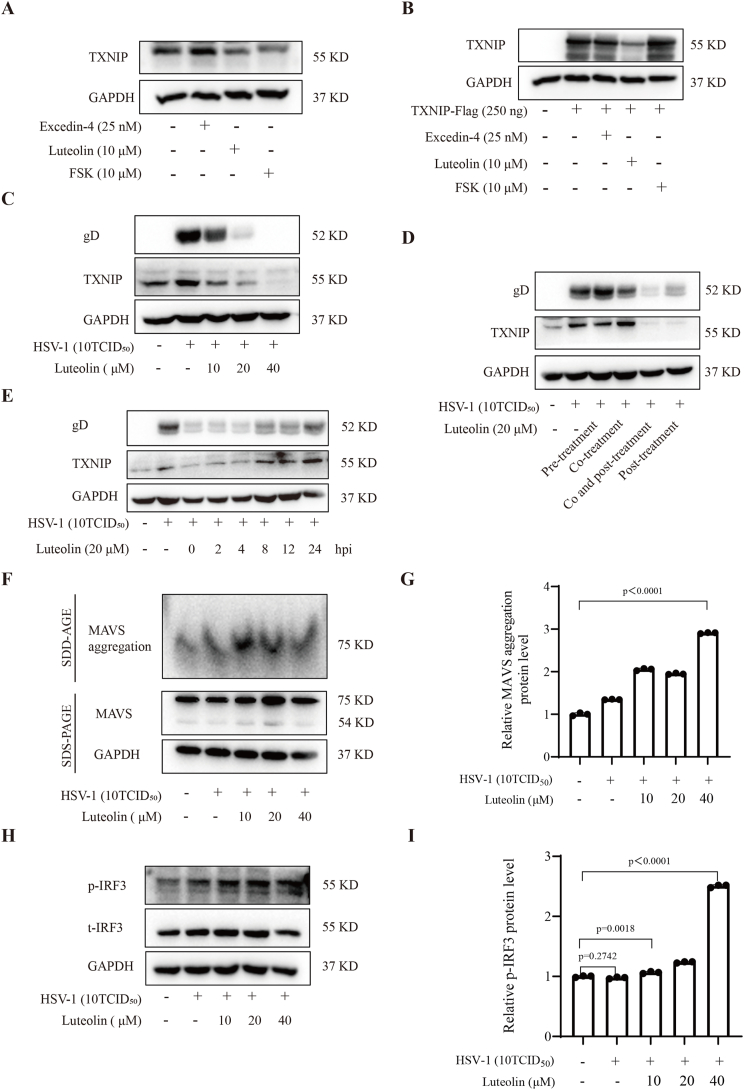
TXNIP degraders significantly inhibit viral replication *in vitro*. (A) 293T cells were treated with the compound, and TXNIP protein expression was assessed after 24 h. (B) TXNIP was overexpressed in 293T cells, followed by treatment with the compound. After 24 h, TXNIP protein levels were measured by Western blotting. (C) 293T cells infected with HSV-1 were treated with varying concentrations of Luteolin (10 μM, 20 μM, and 40 μM), and the expression levels of TXNIP and gD proteins were analyzed by Western blotting. (D) 293T cells were subjected to pre-treatment, co-treatment, post-treatment, or co-post-treatment with Luteolin and HSV-1 virus. After 24 h, TXNIP and gD protein levels were detected by Western blotting. (E) 293T cells were infected with HSV-1, and 20 μM Luteolin was added at 0-, 2-, 4-, 8-, 12- and 24-h post-infection. Protein was collected at 24 h to assess TXNIP and gD expression. (F–I) Under the same conditions as in (C), protein samples were collected, and MAVS aggregation and *p*-IRF3 levels were analyzed by Western blotting. Data are represented as Mean ± SD (n = 3,p < 0.05 was considered as a statistical difference). Statistical significance was determined by one-way ANOVA.

TXNIP is a redox-sensitive protein involved in the regulation of oxidative stress and innate immunity [33]. Given that TXNIP promotes HSV-1 infection by directly interacting with and inhibiting the aggregation of MAVS, this suggests that targeted degradation of TXNIP may represent a promising antiviral strategy. Notably, compounds such as Exendin-4 and forskolin (FSK) have been reported to facilitate TXNIP degradation through PKA Cα-dependent mechanisms, thereby alleviating endoplasmic reticulum (ER) stress-induced β-cell dysfunction and inflammation [35]. Additionally, Luteolin has been shown to inhibit TXNIP expression and reduce ER stress-mediated β-cell apoptosis [36]. Building on these insights, we selected several compounds previously demonstrated to downregulate TXNIP in diabetic models, hypothesizing that they may also exhibit antiviral activity by suppressing TXNIP expression. We first assessed the ability of selected compounds to downregulate TXNIP protein levels in both wild-type and TXNIP-overexpressing 293T cells. Cells were treated with the candidate compounds, and TXNIP levels were evaluated via Western blotting. The results showed that Luteolin significantly reduced TXNIP expression in both normal and TXNIP-overexpressing 293T cells (Fig. 6A-B), identifying it's a solid TXNIP degrader.

Next, we evaluated whether Luteolin-mediated reduction in TXNIP expression translates into antiviral activity against HSV-1. HSV-1-infected 293T cells were treated with increasing concentrations of Luteolin (10 μM, 20 μM and 40 μM), and Western blotting analysis was performed to measure TXNIP and viral gD protein levels. A dose-dependent reduction in both TXNIP and gD protein expression was observed (Fig. 6C), suggesting that Luteolin inhibits HSV-1 replication by downregulating TXNIP. A similar inhibitory effect of Luteolin was also observed during DENV2 infection on viral protein level (Fig. S3E). To determine the timing and stage-specific effects of Luteolin, we performed pre-treatment, co-treatment, and post-treatment of HSV-1-infected cells [[37], [38], [39]]. The results showed that both co-treatment and post-treatment significantly suppressed TXNIP and gD protein levels (Fig. 6D). Time-addition experiments revealed that the antiviral effect of Luteolin was most pronounced when administered within 8 h post-infection. Administration beyond this time point failed to significantly reduce gD or TXNIP expression (Fig. 6E), indicating a time-dependent mechanism that favors early intervention.

These observations underscore the functional correlation between TXNIP expression and HSV-1 replication, reinforcing the concept that TXNIP facilitates viral propagation. To investigate the downstream signaling effects, we examined MAVS aggregation in HSV-1-infected cells treated with increasing doses of Luteolin. SDD-AGE assay revealed that Luteolin enhanced MAVS aggregation in a dose-dependent manner (Fig. 6F-G). Concurrently, we observed elevated *p*-IRF3/t-IRF3 ratios (Fig. 6H-I), indicating enhanced activation of the TBK1-IRF3 pathway. In conclusion, our findings demonstrate that Luteolin exerts antiviral effects by targeting the TXNIP-MAVS-IRF3 signaling axis. Through downregulation of TXNIP, Luteolin restores MAVS aggregation and activation of downstream antiviral signaling, thereby inhibiting viral replication. These results provide strong mechanistic evidence supporting the therapeutic potential of TXNIP-targeting compounds such as Luteolin in the treatment of viral infection.

### 3.7 TXNIP degrader*,* luteolin, shows effective antiviral ability *in vivo*

**Fig. 7:**
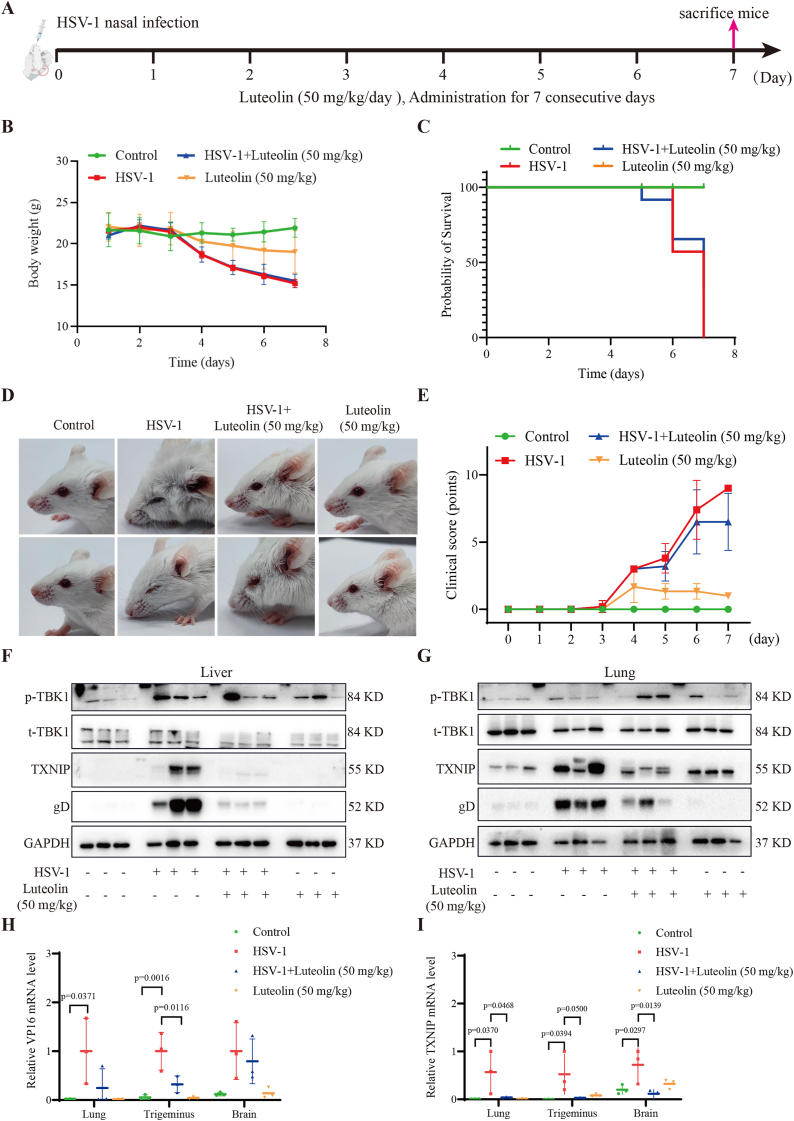
*In vivo* antiviral effect of Luteolin, a TXNIP **degrader.** (A) Schematic representation of the experimental design. BALB/c mice were intranasally inoculated with HSV-1 (1 × 10^7^ PFU) under isoflurane anesthesia. Luteolin (50 mg/kg/day) was administered via oral gavage once daily for 7 consecutive days. Surviving mice were euthanized on day 7 post-infection for tissue analysis. (B) Daily monitoring of body weight changes in each group (n = 5 per group). (C) Kaplan-Meier survival curves of mice in each experimental group (n = 5). (D) Representative images of ocular lesions observed in the different treatment groups on day 7 post-infection. (E) Mouse body condition score. (F) Western blotting analysis of HSV-1 glycoprotein D (gD), TXNIP, and phosphorylated TBK1 (*p*-TBK1) expression in liver tissues. (G) Western blotting analysis of gD, TXNIP, and *p*-TBK1 expression in lung tissues. (H) RT-qPCR analysis of HSV-1 VP16 mRNA levels in lung, trigeminal ganglion and brain tissues. (I) RT-qPCR analysis of TXNIP mRNA levels in lung, trigeminal ganglion and brain tissues. Data are represented as Mean ± SD from (p < 0.05 was considered as a statistical difference). For *in vivo* experiments, n = 5 for each group. Statistical significance was determined by one-way ANOVA.

Cell-based studies elucidate the molecular mechanism underlying the antiviral activity of Luteolin via modulation of the TXNIP-MAVS-IRF3 axis. However, *in vitro* models cannot fully recapitulate the complex physiological conditions present *in vivo*. To further validate the antiviral efficacy of Luteolin and its regulatory role in innate immune signaling under physiological conditions, we performed a series of experiments using a murine model of HSV-1 infection (Fig. 7A). In this study, six-week-old male BALB/c mice were randomly assigned to four groups: control (n = 5), HSV-1 infection (n = 5), HSV-1 + Luteolin (50 mg/kg, n = 5), and Luteolin-only (50 mg/kg, n = 5). HSV-1 was diluted to a concentration of 1 × 10^7^ PFU/mL in serum-free DMEM, and each mouse received a 20 μL intranasal inoculation following anesthesia with isoflurane (2.5 %–5.0 %). Luteolin was administered via oral gavage daily for seven consecutive days. Body weight was monitored daily, and mice were sacrificed on day 7 post-infection for tissue collection and further analysis (Fig. 7B). While Luteolin + HSV infection did not improve body weight loss, which maybe caused by the weigth control effect of Luteolin [40]. Survival analysis revealed a slight improvement in the Luteolin-treated group compared to the HSV-1-infected group (Fig. 7C). Additionally, among surviving animals, ocular lesions were ameliorated in the Luteolin-treated group (Fig. 7D). The pathological scoring of mice showed that Luteolin significantly improved the pathological conditions caused by HSV-1 infection (Fig. 7E). Western blotting analysis of lung and liver tissues showed that Luteolin treatment reduced the expression of HSV-1 glycoprotein D (gD) and TXNIP compared to untreated infected mice. Furthermore, the *p*-TBK1/t-TBK1 ratio was moderately elevated in the treatment group, indicating partial restoration of TBK1 activity (Fig. 7F–G). To further assess viral replication and TXNIP expression, RT-qPCR was performed on RNA isolated from lung, trigeminus ganglion and brain tissue. The results revealed consistent downregulation of HSV-1 VP16 mRNA and *TXNIP* transcripts in multiple tissues of Luteolin-treated mice compared to untreated infected controls (Fig. 7H–I). Taken together, these *in vivo* results confirm that Luteolin exhibits antiviral activity against HSV-1 infection, likely through modulation of the TXNIP-MAVS-IRF3 signaling axis. These findings provide further evidence supporting the therapeutic potential of TXNIP-targeting compounds for antiviral intervention.

## 4 Discussion

TXNIP is a well-known regulator of cellular redox homeostasis and inflammation [33]. Its upregulation induces oxidative stress and has been implicated in various pathological conditions, including diabetes, sepsis, and kidney disease [41]. In the area of viral infection, one study suggested that TXNIP activated Epstein-Barr virus (EBV) replication by activating NLRP3 pathway [18]. Another study indicated that overexpression of TXNIP reduced influenza virus infection, suggesting that TXNIP is an antiviral gene [14]. Collectively, these findings indicate that the role of TXNIP in viral infection remains incompletely understood and requires further clarification.

Our study systematically explores TXNIP's role and mechanism in HSV-1 infection, with findings extended to Dengue virus, elucidating how TXNIP modulates the host antiviral immune response by regulating MAVS aggregation and the TBK1-IRF3 signaling pathway. Our results reveal that TXNIP plays a critical role in HSV-1 infection. Following HSV-1 infection, intracellular TXNIP levels increase. TXNIP specifically interacts with the 1–180 region of MAVS via its 174–300 amino acid domain, inhibiting MAVS oligomerization on mitochondria. This interaction disrupts MAVS-mediated innate immune signaling, ultimately reducing type I interferon secretion-a key component of antiviral immunity. Consequently, these events enable HSV-1 to evade host innate immune surveillance. Notably, HSV-1 infection is significantly suppressed in CRISPR-Cas9-mediated TXNIP-knockout cells or shRNA-mediated TXNIP-knockdown cells, underscoring TXNIP's pivotal role in facilitating viral immune evasion. These findings uncover a novel mechanism by which TXNIP influences viral infection. While our group and others have previously shown that TXNIP promotes inflammation by regulating the NLRP3 inflammasome [31,42,43], this study highlights a novel interaction between TXNIP and MAVS, providing new evidence of TXNIP's role in antiviral immunity.

The cGAS-STING pathway is a key mechanism for sensing DNA during HSV-1 infection, triggering robust innate immune responses. However, HSV-1 has evolved multiple strategies to evade host immunity and enhance its infectivity [44]. In contrast to classical innate immune proteins like cGAS-STING, we propose TXNIP as a novel HSV-1 infection escape protein that operates independently of traditional innate immune molecules. Unlike PRRs such as cGAS or STING, which detect 10.13039/100026054DNA, our co-immunoprecipitation assays reveal that TXNIP exhibits a stronger interaction with MAVS, a key adaptor typically associated with RNA virus sensing. Supporting this, Jesper Melchjorsen et al. identified the MDA5-MAVS signaling axis as a primary mediator of HSV-1 recognition [45]. Our findings demonstrate that TXNIP promotes HSV-1 infection by inhibiting MAVS aggregation, thereby disrupting antiviral signaling. These results highlight TXNIP as a novel therapeutic target for combating HSV-1 infection, offering a new perspective on viral immune evasion mechanisms.

Luteolin, a common flavonoid present in edible and medicinal plants across diverse botanical families (e.g., *Lonicera japonica* and *Chrysanthemum morifolium*), exhibits antimutagenic, antioxidant, and antitumor activities characteristic of plant-derived flavonoids [46]. It is incorporated in multiple clinically used Chinese patent medicines, such as Lanqin Oral Liquid containing 0.82–1.15 mg/g Luteolin, though its suboptimal pharmacokinetics currently preclude standalone therapeutic use. Substantial evidence supports Luteolin's broad antiviral efficacy, inhibits influenza virus replication [47], dengue virus [48], EV71 [49] and HSV-1 ^30^. While our study identified Luteolin as an effective TXNIP degrader [36], which markedly reducing TXNIP protein levels *in vitro* and *in vivo*. In HSV-1-infected mice, Luteolin treatment curbed virus-induced cell death, consistent with prior studies [30]. We also observed significant TXNIP degradation in liver and lung tissues, coupled with increased TBK1 phosphorylation—a key indicator of MAVS activation. These findings show that Luteolin suppresses HSV-1 infection in animal models, mirroring cellular results. It is worth noting that administration of Luteolin led to a reduction in body weight in mice, which may be associated with its role in modulating metabolic function, potentially through the enhancement of pancreatic islet cell activity and regulation of overall metabolic levels [36]. Notably, Luteolin treatment alleviated HSV-1-induced weight loss, as treated mice maintained a higher body weight compared to those in the HSV-1-infected group, suggesting that Luteolin can counteract virus-induced weight reduction. Similarly, other studies have reported that Luteolin has only a limited effect on restoring body weight following HSV-1 infection [30]. Taken together, these findings suggest that while Luteolin possesses intrinsic weight-reducing properties due to its metabolic effects, it can also attenuate virus-induced weight loss. Therefore, although Luteolin alleviates HSV-1 infection, its therapeutic benefits may not be fully reflected in body weight measurements alone.

Dengue virus, a common mosquito-borne single-stranded positive-sense RNA virus in our laboratory and a pathogen in urgent need of antiviral drug research [28]. We found that dengue virus infection also promotes TXNIP overexpression, and TXNIP overexpression significantly enhances dengue virus infection. Conversely, TXNIP knockout via CRISPR-Cas9 or knockdown via shRNA inhibits dengue virus infection. Minhua Peng et al. have also reported the anti-dengue efficacy of the TXNIP degrader Luteolin [48], demonstrating that TXNIP intervention indeed suppresses dengue virus infection and providing a new molecular target for dengue virus prevention and treatment. Compared with DNA viruses, upon recognition of viral RNA by the PRR molecule, such as RIG-I, RNA viruses further activate the MAVS-TBK1-mediated innate immune signaling pathway to exert antiviral effects. This study also indicated the crucial role of TXNIP in the RNA virus's infection.

Although this study elucidated the mechanism of TXNIP in viral infections and validated the antiviral efficacy of Luteolin, several questions remain to be addressed. First, the differential roles of TXNIP in DNA and RNA virus infections warrant further investigation. DNA and RNA viruses exhibit significant differences in their infection mechanisms and host immune responses [50]. While this study primarily focused on the role of TXNIP in HSV-1 and DENV2 infections, future research could explore its involvement in other viral infections. Notably, some studies indicate that TXNIP is significantly upregulated following influenza virus infection, and its overexpression inhibits viral replication [14], which contrasts with our findings. The mechanisms underlying these divergent effects deserve further investigation. By comparing the regulatory roles of TXNIP across different viral infections, a theoretical foundation for developing broad-spectrum antiviral drugs could be established. In addition, our current detection was only performed in cell lines and primary blood monocytes, further studies should validate these findings in other primary cells, such as human primary cells.

In summary, this study highlights the critical role of TXNIP in HSV-1 and DENV2 infections and elucidates the molecular mechanism by which TXNIP attenuates the host's antiviral immune response by inhibiting MAVS aggregation and the TBK1-IRF3 signaling pathway. Although Luteolin exhibits complex pharmacological effects [30,48,51,52], our results assign a new function to Luteolin as a TXNIP degrader, demonstrates significant antiviral efficacy in both cellular and animal models, providing a strong theoretical foundation for the development of TXNIP-based antiviral therapies. These studies will offer new insights into the regulatory mechanisms of TXNIP in antiviral immunity and lay the groundwork for the development of Luteolin as a broad-spectrum antiviral drug.

## CRediT authorship contribution statement

**Ruilin Chen:** Visualization. **Wenyu Wu:** Writing – review & editing, Writing – original draft. **Jiajia Han:** Visualization. **Qiyun Lei:** Visualization. **Jiawan Chen:** Data curation. **Dan Guo:** Data curation. **Chan Wang:** Visualization. **Xiaodong Tang:** Visualization. **Min Zou:** Visualization. **Shuwen Liu:** Conceptualization. **Xingang Yao:** Writing – review & editing, Writing – original draft, Funding acquisition, Data curation, Conceptualization.

## Fundings

This work was supported by the 10.13039/501100001809National Natural Science Foundation of China (82373873), and 10.13039/501100021171Guangdong Basic and Applied Basic Research Foundation (2023A1515030057), and Key-Area Research and Development Program of Guangdong Province (2023B1111050008).

## Declaration of competing interest

The authors declare that they have no known competing financial interests or personal relationships that could have appeared to influence the work reported in this paper.

## Figures and Tables

**Figure:**
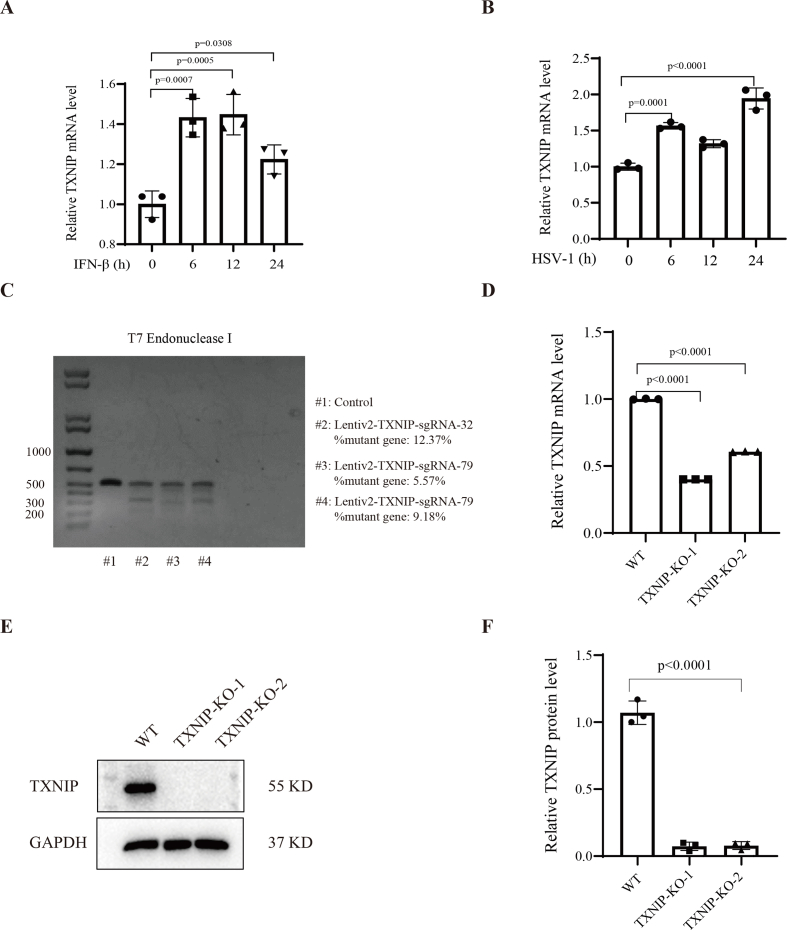
Fig. S1Generation of TXNIP knockout cell lines. (A) The expression levels of TXNIP gene in 293T cells treated with IFN-β at different time points were detected by RT-qPCR. (B) The expression levels of TXNIP gene in 293T cells infected with HSV-1 at different time points were detected by RT-qPCR. (C) The gene editing efficiency of TXNIP-sgRNA was assessed using the T7EI assay. (D) The mRNA level of TXNIP in TXNIP-KO cells was measured by RT-qPCR. (E-F) The protein level of TXNIP in TXNIP-KO cells was analyzed by Western blotting. Data are represented as Mean ± SD (n = 3,p < 0.05 was considered as a statistical difference). Statistical significance was determined by one-way ANOVA.

**Figure:**
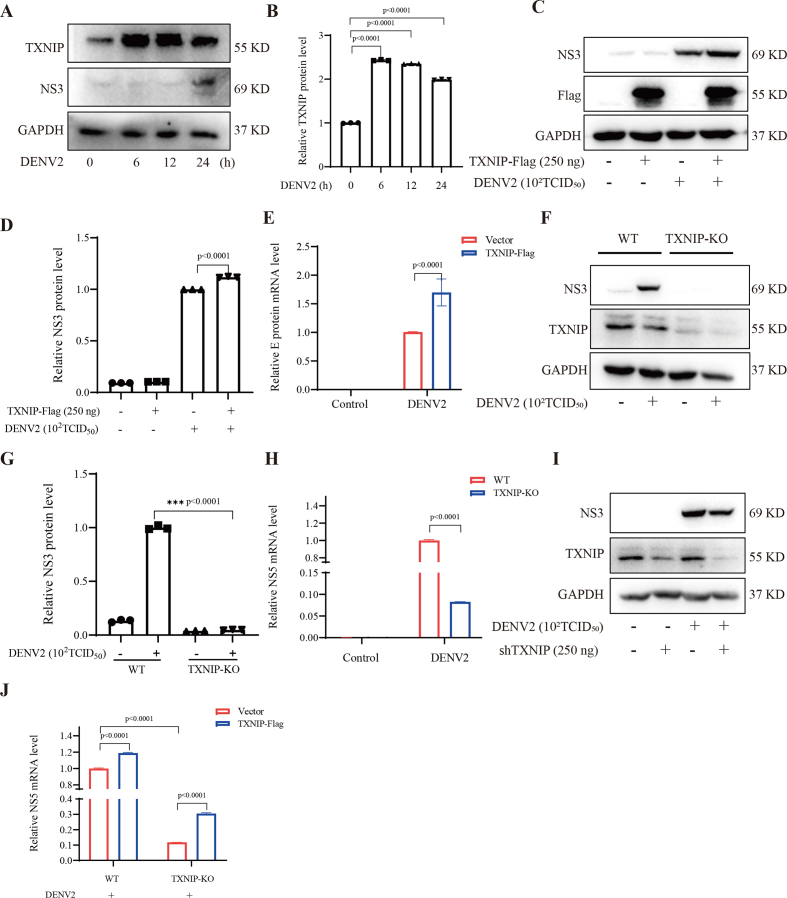
Fig. S2TXNIP participated in DENV2 infection. (A) TXNIP protein expression levels in blood monocytes cell were detected by Western blotting after infection with DENV2 at 0-, 6-, 12-, and 24- hours. (B) Quantitative TXNIP protein levels analysis of (A). (C) 293T cells were transfected with either an empty vector (pcDNA3.1a) or TXNIP-Flag for 24 h, followed by DENV2 infection for 48 h. The expression level of NS3 protein was then assessed by Western blotting. (D) Quantitative analysis of NS3 protein levels from (C). (E) The mRNA expression level of the E protein was measured by RT-qPCR under the same condition of (C). (F) 293T-WT and TXNIP-KO cells were infected with DENV2, and after 48 h, RNA and proteins were extracted. The expression of the viral protein NS3 was detected by Western blotting. (G) Quantitative TXNIP protein levels analysis of (F). (H) The mRNA expression of the viral gene NS5 was quantified by RT-qPCR under the same condition of (F). (I) 293T cells were transfected with either an empty vector (pLKO.1 vector) or TXNIP-shRNA for 24 h, followed by DENV2 infection for 48 h. The expression levels of NS3 and TXNIP proteins were analyzed by Western blotting. (J) 293T-WT and TXNIP-KO cells were overexpressed with TXNIP, followed by DENV2 infection. The mRNA level of the viral gene NS5 was measured by RT-qPCR. Data are represented as Mean ± SD (n = 3,p < 0.05 was considered as a statistical difference). Statistical significance was determined by one-way ANOVA or two-sided Student's t-test as appropriate.

**Figure:**
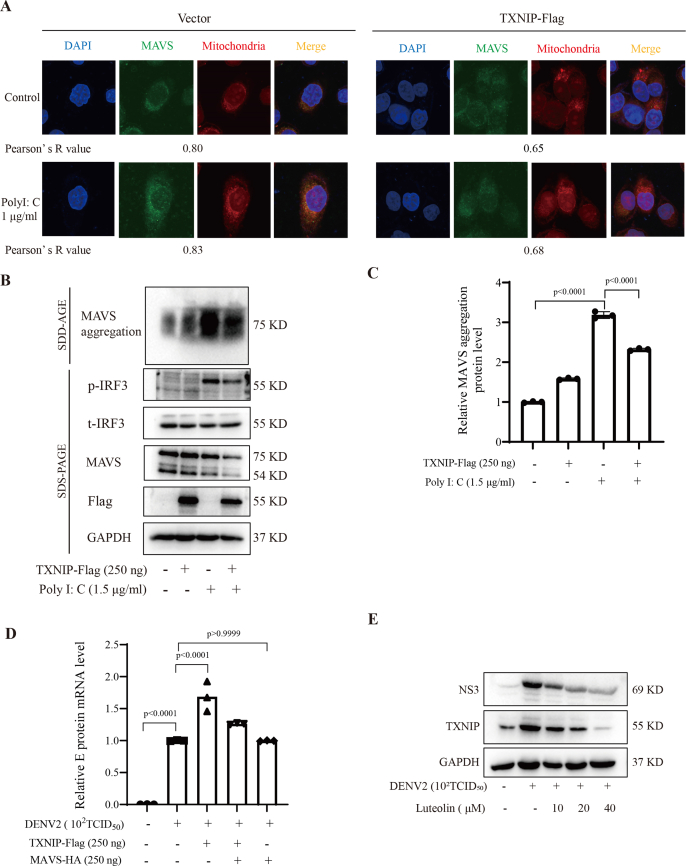
Fig. S3The effect of TXNIP on the MAVS aggregation. (A) Immunofluorescence staining was performed on Poly(I:C)-stimulated HeLa cells with TXNIP overexpression, and mitochondrial MAVS aggregation was analyzed by confocal microscopy 24 h post-stimulation. (B) Western blotting analysis of MAVS aggregation and *p*-IRF3/t-IRF3 in TXNIP-overexpressing 293T cells were performed using SDD-AGE (top) and SDS-PAGE (bottom) after Poly (I: C) stimulation. (C) The MAVS aggregation quantitative result of (B). (D) 293T cells were transfected or co-transfected with TXNIP and MAVS, followed by DENV2 infection. The mRNA levels of the E protein were quantified using RT-qPCR. (E) 293T cells infected with DENV2 were treated with varying concentrations of luteolin (10 μM, 20 μM and 40 μM), and the expression levels of TXNIP and NS3 proteins were analyzed by Western blotting. Data are represented as Mean ± SD (n = 3, p < 0.05 was considered as a statistical difference). Statistical significance was determined by one-way ANOVA.

## Data Availability

Data will be made available on request.
