# Supplementary material for: TXNIP promotes viral replication by disrupting MAVS-mediated antiviral signaling and serves as a therapeutic target for antiviral therapy
Source: Redox Biol. 2025 Jul 5;85:103756. doi: 10.1016/j.redox.2025.103756 (PMC12273484; doi:10.1016/j.redox.2025.103756)
Supplement: Multimedia component 1 [file mmc1.docx]

**Supplemental figures**


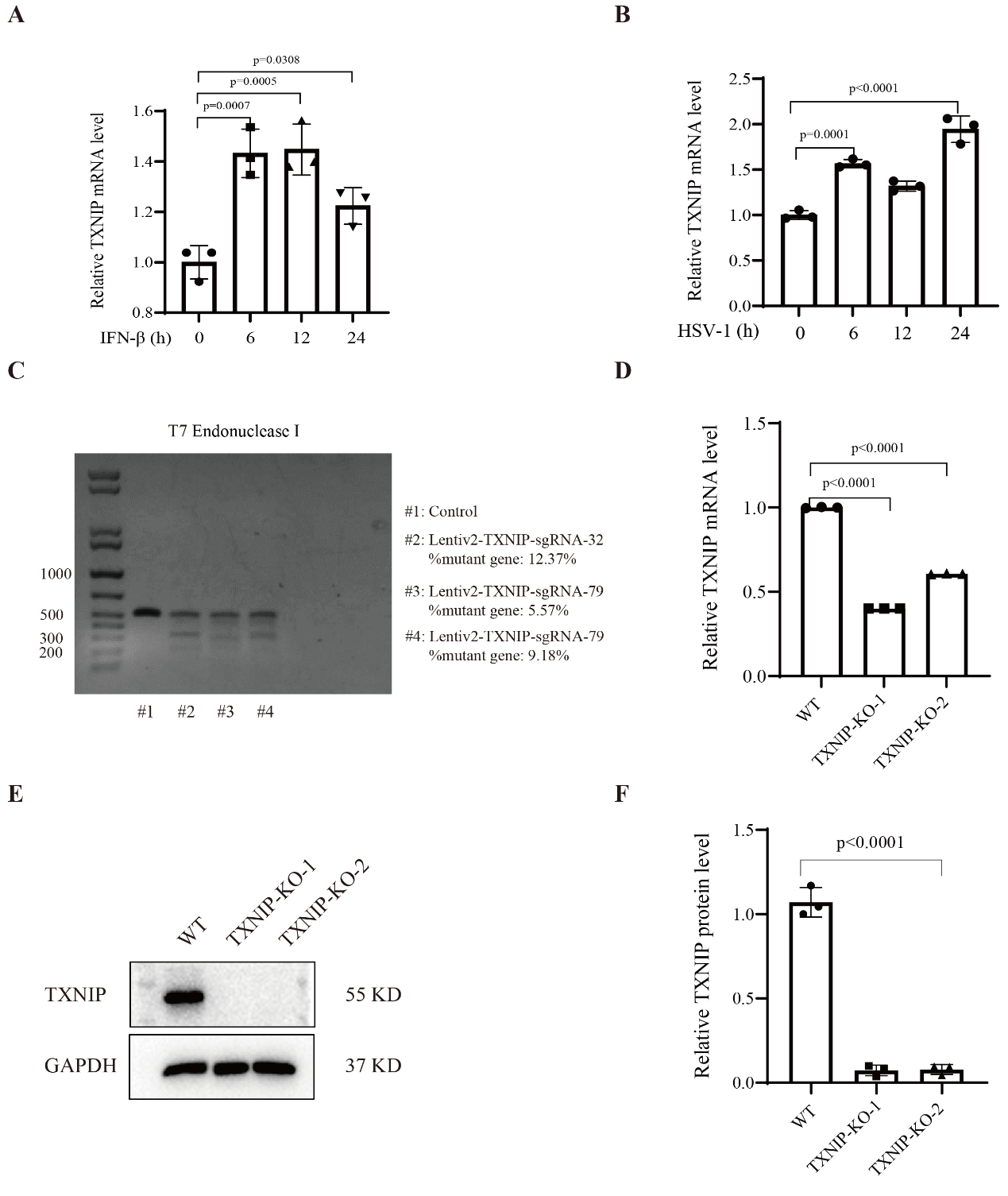


Fig. S1 Generation of TXNIP knockout cell lines. (A) The expression levels of TXNIP gene in 293T cells treated with IFN-β at different time points were detected by RT-qPCR. (B) The expression levels of TXNIP gene in 293T cells infected with HSV-1 at different time points were detected by RT-qPCR. (C) The gene editing efficiency of TXNIP-sgRNA was assessed using the T7EI assay. (D) The mRNA level of TXNIP in TXNIP-KO cells was measured by RT-qPCR. (E-F) The protein level of TXNIP in TXNIP-KO cells was analyzed by Western blotting. Data are represented as Mean ± SD (n=3，p<0.05 was considered as a statistical difference). Statistical significance was determined by one-way ANOVA.


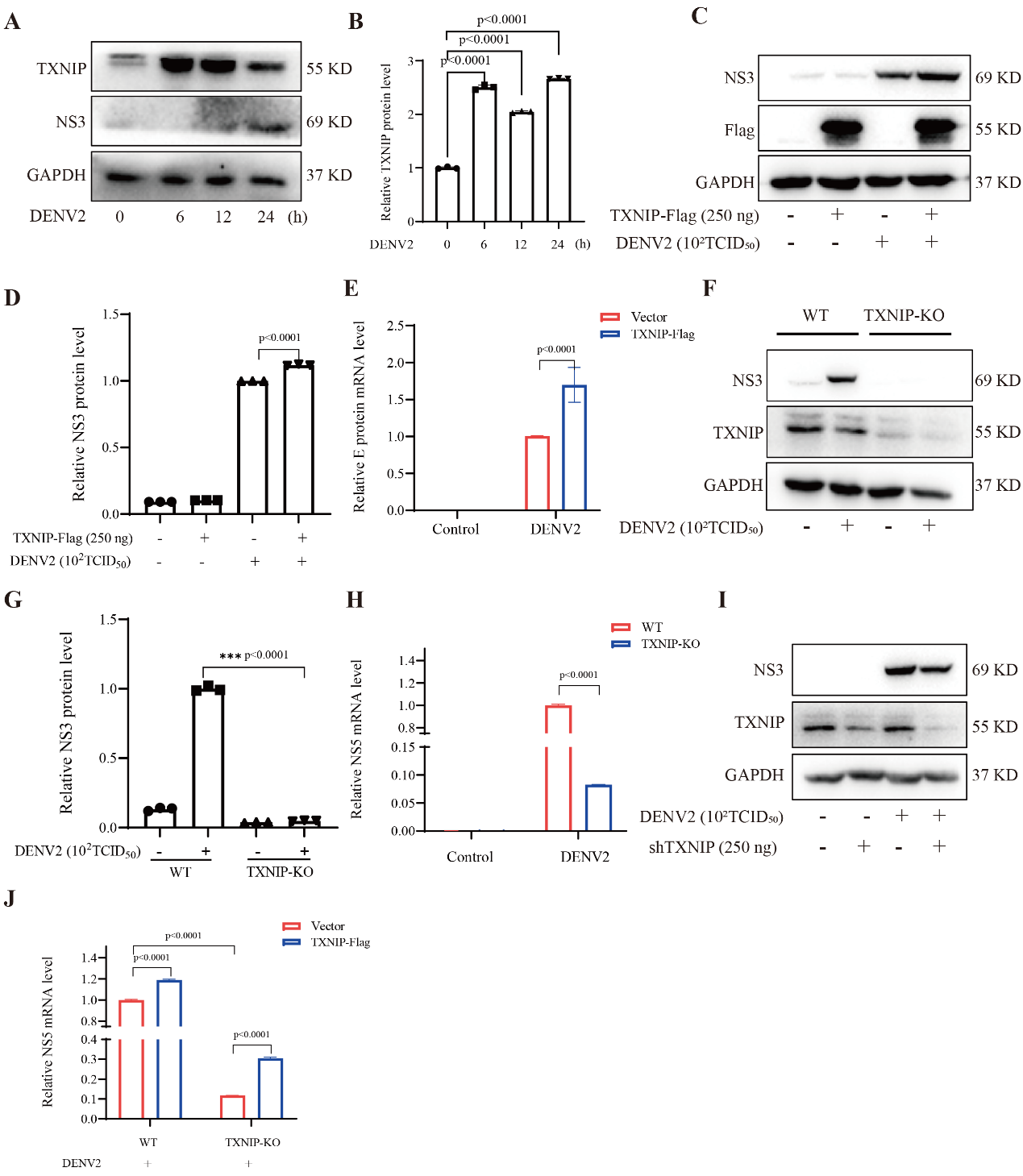


Fig. S2 TXNIP participated in DENV2 infection. (A) TXNIP protein expression levels in blood monocytes cell were detected by Western blotting after infection with DENV2 at 0-, 6-, 12-, and 24- hours. (B) Quantitative TXNIP protein levels analysis of (A). (C) 293T cells were transfected with either an empty vector (pcDNA3.1a) or TXNIP-Flag for 24 hours, followed by DENV2 infection for 48 hours. The expression level of NS3 protein was then assessed by Western blotting. (D) Quantitative analysis of NS3 protein levels from (C). (E) The mRNA expression level of the E protein was measured by RT-qPCR under the same condition of (C). (F) 293T-WT and TXNIP-KO cells were infected with DENV2, and after 48 hours, RNA and proteins were extracted. The expression of the viral protein NS3 was detected by Western blotting. (G) Quantitative TXNIP protein levels analysis of (F). (H) The mRNA expression of the viral gene NS5 was quantified by RT-qPCR under the same condition of (F). (I) 293T cells were transfected with either an empty vector (pLKO.1 vector) or TXNIP-shRNA for 24 hours, followed by DENV2 infection for 48 hours. The expression levels of NS3 and TXNIP proteins were analyzed by Western blotting. (J) 293T-WT and TXNIP-KO cells were overexpressed with TXNIP, followed by DENV2 infection. The mRNA level of the viral gene NS5 was measured by RT-qPCR. Data are represented as Mean ± SD (n=3，p<0.05 was considered as a statistical difference). Statistical significance was determined by one-way ANOVA or two-sided Student's t-test as appropriate.


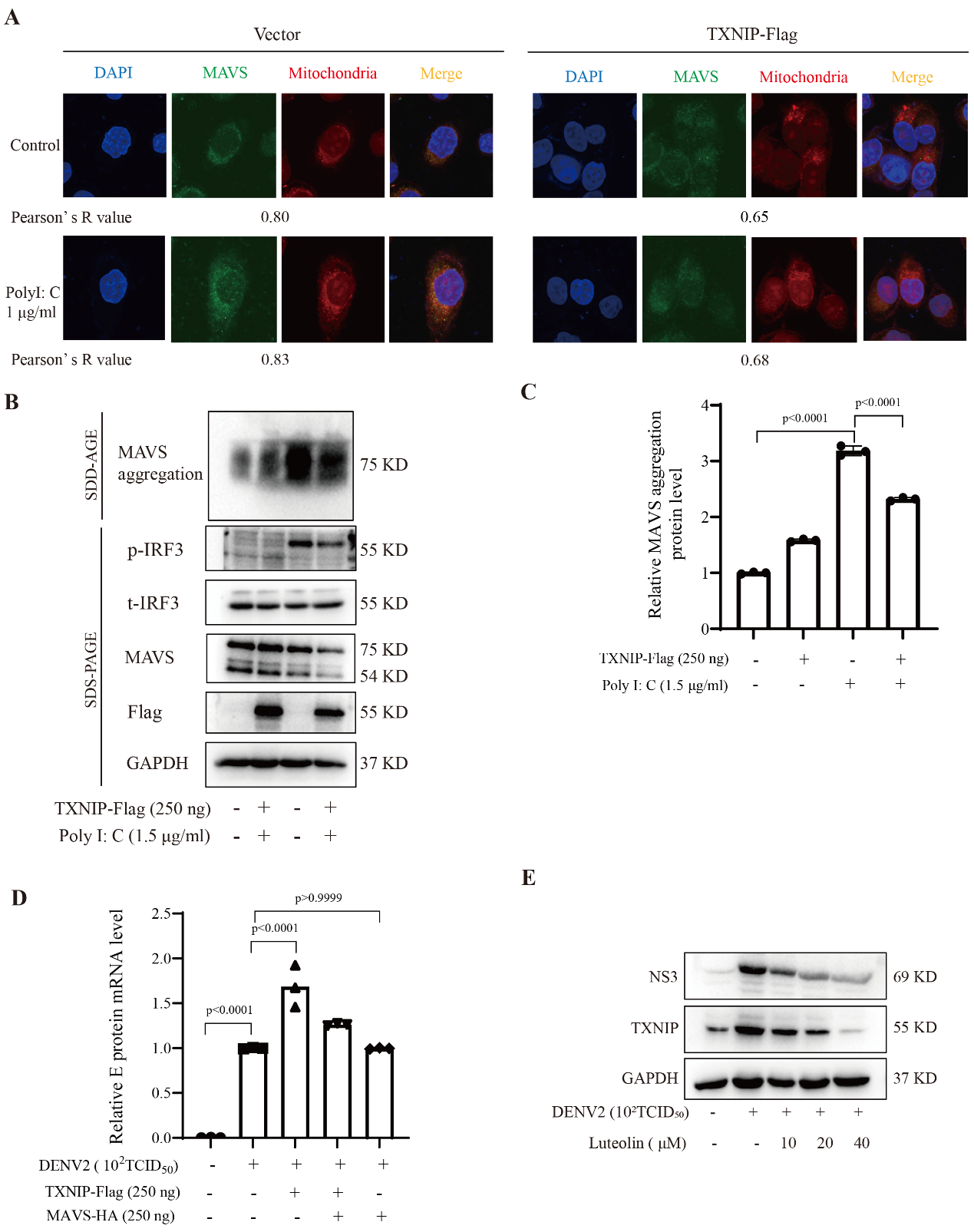


Fig. S3 The effect of TXNIP on the MAVS aggregation. (A) Immunofluorescence staining was performed on Poly(I:C)-stimulated HeLa cells with TXNIP overexpression, and mitochondrial MAVS aggregation was analyzed by confocal microscopy 24 hours post-stimulation. (B) Western blotting analysis of MAVS aggregation and p-IRF3/t-IRF3 in TXNIP-overexpressing 293T cells were performed using SDD-AGE (top) and SDS-PAGE (bottom) after Poly (I: C) stimulation. (C) The MAVS aggregation quantitative result of (B). (D) 293T cells were transfected or co-transfected with TXNIP and MAVS, followed by DENV2 infection. The mRNA levels of the E protein were quantified using RT-qPCR. (E) 293T cells infected with DENV2 were treated with varying concentrations of luteolin (10 μM, 20 μM and 40 μM), and the expression levels of TXNIP and NS3 proteins were analyzed by Western blotting. Data are represented as Mean ± SD (n=3, p<0.05 was considered as a statistical difference). Statistical significance was determined by one-way ANOVA.
